# The Mechanism Action of German Chamomile (*Matricaria recutita L.*) in the Treatment of Eczema: Based on Dose–Effect Weight Coefficient Network Pharmacology

**DOI:** 10.3389/fphar.2021.706836

**Published:** 2021-09-30

**Authors:** Wenfei Wang, Yichun Wang, Junbo Zou, Yanzhuo Jia, Yao Wang, Jia Li, Changli Wang, Jing Sun, Dongyan Guo, Fang Wang, Zhenfeng Wu, Ming Yang, Lei Wu, Xiaofei Zhang, Yajun Shi

**Affiliations:** ^1^ Department of Pharmaceutics, College of Pharmacy, Shaanxi University of Chinese Medicine, Xianyang, China; ^2^ Department of Pharmaceutics, The Key Laboratory of Basicand New Drug Research of Traditional Chinese Medicine, Shaaxi University of Chinese Medicine, Xianyang, China; ^3^ Department of Pharmaceutics, Key Laboratory of Modern Preparation of Traditional Chinese Medicine, Ministry of Education, Jiangxi University of Chinese Medicine, Nanchang, China; ^4^ Henan Feinari Aromatic Biotechnology Co., Ltd, Zhumadian, China

**Keywords:** German chamomile, eczema, weight coefficient, molecular docking, mechanism verification

## Abstract

To determine the active ingredients in German chamomile volatile oil and the mechanism of action in the treatment of eczema, this study used two parameters (ingredient content and oil–water partition coefficient) and established a new network pharmacology method based on the dose–effect weight coefficient. Through the new network pharmacology method, we found that German chamomile volatile oil regulated T-cell lymphatic subpopulations to inhibit the Th17 cell differentiation signaling pathway. This resulted in a reduction of interleukin 17 (IL-17), thereby inhibiting the activation of the nuclear factor kappa beta (NF-κB) and MAPK pathways, decreasing the secretion of the pro-inflammatory factors (tumor necrosis factor alpha (TNF-α) and interleukin 6 (IL-6)), and reducing inflammation. In this study, a new dose–effect relationship synergistic network pharmacology method was established to provide a new method for the screening of effective ingredients and pathways of drugs, and to provide a basis for the follow-up studies of German chamomile volatile oil in the treatment of eczema.

## Introduction

Eczema is an inflammatory skin disease usually accompanied by infiltration, hypertrophy, and severe itching. Studies have shown that the occurrence of eczema is related to many factors, including immune, environmental, and genetic factors, as well as infection. Eczema has a long course and recurs easily, seriously affecting the quality of life. At present, antihistamines, anti-allergic drugs, and glucocorticoids are commonly used in the clinic. However, these drugs only temporarily relieve symptoms and may result in adverse reactions with long-term use. Thus, it is important to develop natural drugs with minimal side effects for the effective treatment of eczema.

**GRAPHICAL ABSTRACT:**
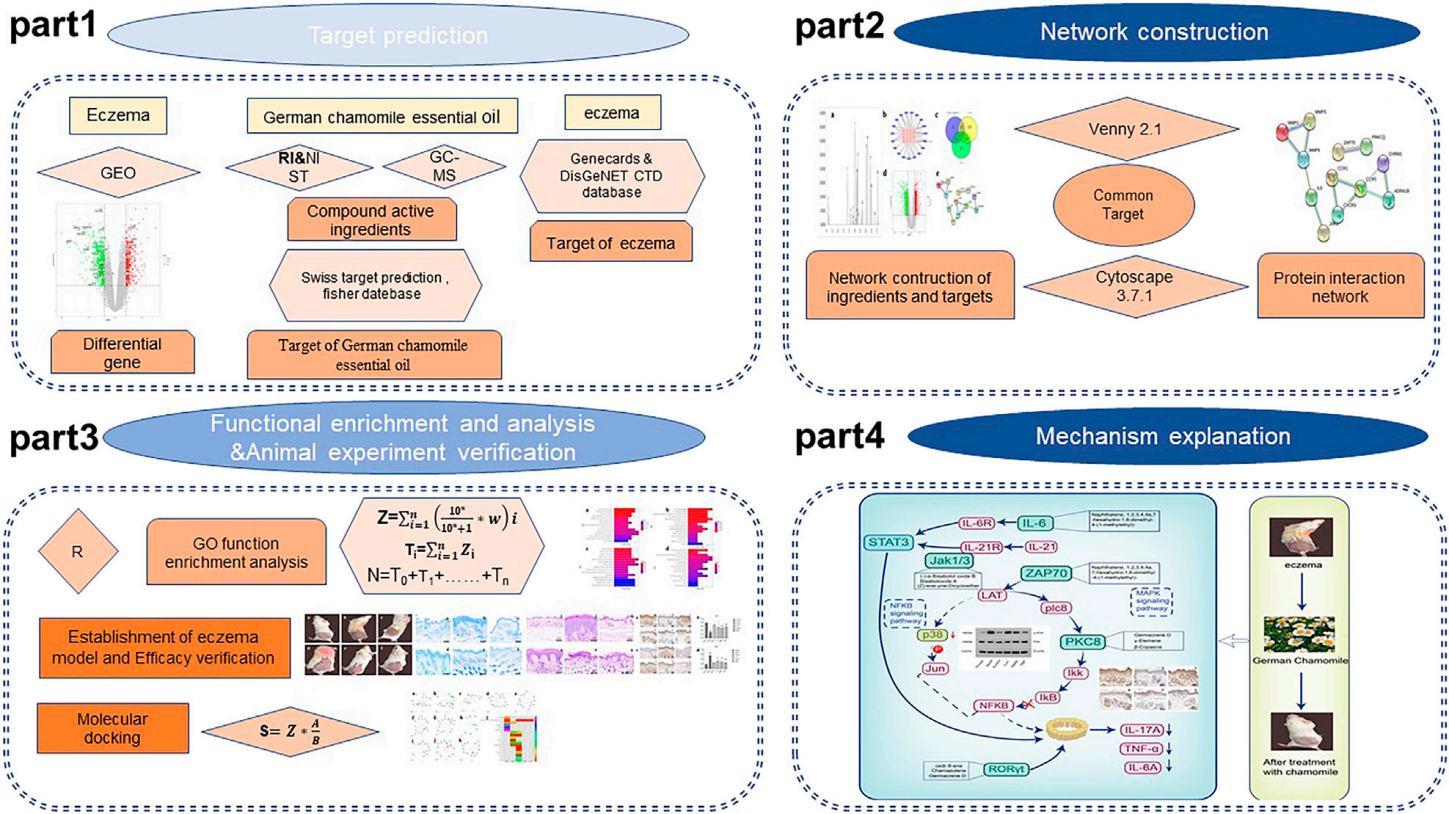


Chamomile (*Matricaria recutita L.*) is one of the oldest aromatic plants, and the flower and essential oils have been shown to exhibit anti-inflammatory and antispasmodic effects. Chamomile is widely used in food, cosmetics, disinfectants, and medicines. Therefore, it is a medicinal plant with great developmental potential. German chamomile belongs to the family Compositae, and its volatile oil is often used in cosmetic care to eliminate inflammation, relieve skin irritation, and reduce redness and swelling of the skin. This oil has been used for healing skin wound and exhibits antibacterial, anti-inflammatory, and antioxidant activities. The cyclic ethers, flavonoids, and total volatile oils in chamomile have also been shown to exhibit an inhibitory effect on fungal growth. Polysaccharides isolated from its inflorescence also exhibit anti-inflammatory activity. Several studies have reported significant effects of German chamomile volatile oil in the treatment of eczema (Anderson et al., 2000; Patzelt-Wenczler and Ponce-Pöschl, 2000; Arsić et al., 2011; El-Salamouni et al., 2020). However, neither the active components nor the pharmacological mechanism of its volatile oil is clear. This study used an eczema mouse model to explore the therapeutic effects of German chamomile volatile oil on eczema and to determine the therapeutic mechanism. The results provide a reference for further elucidation of the mechanism of action in future studies.

Network pharmacology explains the development of a disease via establishment of a database, network, network analysis, and experimental verification. It also attempts to determine the interaction between drugs and the body and to guide the discovery of new drugs. However, at present, most network pharmacology research treats the content of all components equally, ignoring the effects of both drug content and concentration on efficacy. This may lead to the identification of components with low content as key components. At the same time, components with high content that play a key role in treatment are often ignored. As a result, the existing network pharmacology research has been unable to identify ingredients and the mechanism of action that are responsible for observed effects. Network pharmacology generally uses oral bioavailability (OB) and drug-like (DL) property to screen for active ingredients in drugs. However, this method cannot be applied to predict transdermal drug delivery, resulting in the inability to obtain the targets and mechanisms of transdermal drugs. To address the above issues, a new method, “dose–effect weight coefficient method and network pharmacology” method, has been established, which can be used for transdermal drugs to mine components, targets, and pathways. Component content and oil–water partition coefficient, and molecular docking score are combined to obtain the theoretical transdermal absorption content of each component. Using this method, the contribution score of the corresponding “weight coefficient” is established, compared with the signaling pathways enriched by traditional network pharmacology, and the key pathways are selected. For the purpose of this study, a mouse eczema model was established using dinitrochlorobenzene (DNCB) to explore the mechanism of action and to verify the efficacy of German chamomile volatile oil in the treatment of eczema.

## Materials and Methods

### Determination of Chemical Composition of Volatile Oil From German Chamomile

German chamomile essential oil (purchased from Henan Feinari Aromatic Biotechnology Co., Ltd,) was identified by gas chromatography–mass spectrometery (GC–MS) analysis (Tai et al., 2020). The chromatographic conditions were HP-5 MS capillary column (30 m × 250 μm x 0.25 µm), carrier gas He, carrier gas as shunt mode, injection of 1.0 μL, and a column flow rate of 1 ml/min. The starting temperature was 40°C, and the temperature was increased to 250°C at a rate of 6°C/min. The mass spectrometry conditions were full scan mode, ionization source EI, an ionization energy of 70 eV, transmission line temperature 280°C, ion source temperature 230°C, quadrupole temperature 150°C, solvent delay time 3 min, and scanning quality range of 20–450 amu (Fu et al., 2018).

### Identification of Essential Oil

The data were processed using data analysis software. The NIST standard spectral database was used to search, and the components were screened according to the matching degree, retention index, and related literature. The retention index was defined as the retention time of n-alkanes (C8–C40) under the same gas chromatographic conditions. According to the retention index of n-alkanes, the retention index of German chamomile volatile oil was as follows:

$$RI=100\text{Z}+\frac{100[\text{tR}\left(\text{X}\right)-\text{tR}(\text{Z})]}{\text{tR}\left(\text{Z}+1\right)-\text{tR}\left(\text{Z}\right)}$$ (1-1)

where tR is the retention time, X is the compound to be analyzed, and Z and Z+ 1 are the number of carbon atoms of the two n-alkanes before and after the analyte, namely, tR (Z) < tR (x) < tR (Z+ 1) (Yuandong et al., 2017).

### Pharmacological Analysis of the Ingredient-Target Network

#### Determination of Volatile Oil of German Chamomile Targets

The Swiss Target Prediction database (Le-qi et al., 2020) and meta TarFisher databases were used to predict the volatile oil targets of German chamomile, and the targets of the active components of German chamomile volatile oil were obtained.

#### Acquisition of Eczema Disease Targets

Taking “eczema” as the keyword and setting the species as humans, we searched the GeneCards database (Liu et al., 2020), DisGeNET database (Wu et al., 2020), OMIM database (Kui et al., 2021), and Comparative Toxicogenomics Database (CTD) (Tianmu et al., 2021) to identify the qualified information of genes and target proteins related to eczema.

#### Gene Expression Omnibus (GEO)database Chip Differential Gene Verification

Based on the GEO database GSE57225 chip detection data (Liang et al., 2021), the data set included 23 patients and 17 normal control samples. The differential gene expression was analyzed using Limma software package 15 of R. The screening criteria were | logFC | > 1.2 and *p* < 0.05.

#### Construction of the Compound Composition–Target Network

The volatile oil target, eczema target, and GEO differential gene of German chamomile were intersected by using Venny 2.1.0 (https://bioinfogp.cnb.csic.es/tools/venny/index.html). The active components of German chamomile volatile oil and the three overlapping genes were introduced into Cytoscape 3.7.1 (Shi et al., 2020) to construct the compound component–target network, Construction and analysis of the protein–protein interaction (PPI) 1.3.5 (Xu et al., 2020) network.

The potential targets of the active components of volatile oil, the disease targets of eczema, and the differential genes mined by GEO were integrated, intersectedm and introduced into the STRING database (Sang et al., 2020). The minimum interaction threshold was set to “highest confidence.” The free protein was hidden, and the protein relationship map was obtained. Results were imported into Cytoscape 3.7.1 software, and the interaction network was drawn and analyzed.

### Establishment of the Weight Coefficient

The existing network pharmacology studies usually use oral bioavailability (OB) and drug-like (DL) property to screen for active ingredients in drugs. However, this method cannot be used to predict transdermal drug delivery, especially transdermal drug targets and mechanisms. To better explain the relationship between the transdermal absorption components and activity of chamomile, we introduced the log P value (an important parameter in the measurement of transdermal drug absorption) to convert the transdermal absorption rate and correlate the two to establish a relationship:

$$\log P=log\frac{Coil}{Cwater}=n$$

CoilCwater=10^n

CoilCoil+Cwater=10^n10^n+1.

Because the drug application is percutaneous, the concentration of n-octanol (oil) indicates 100% complete absorption of the drug, and C water can indicate the part of the drug that has not been absorbed. Therefore, Coil/(Coil + C water) represents the absorption rate of volatile oil.

In order to better explain the relationship between oil–water partition coefficient, relative content, and active components in pharmacological mechanism, we correlate the three:

Z=∑i=1n(10^n10^n+1⋅w)i (1-2)

$$T_{i}=\sum_{i=1}^{n}Z_{i}$$ (1-3)

$$\mathrm{N=}T_{0}+T_{1}+\ldots \ldots +T_{n}$$ (1-4)

In the formula, Z is the calculated score of each active component, T represents the sum of the fractions of the related active components contained in each target, and N is the sum of the target fractions contained in each pathway.

### Gene Ontology–Biological Process Enrichment Analysis and Kyoto Encyclopedia of Genes and Genomes Pathway Enrichment Analysis

To further clarify the gene function of German chamomile volatile oil and the role of potential signaling pathway targets in eczema, profile in the Rstudio was used to analyze the key targets in GO–BP, the basic biological processes and pathways were analyzed by the KEGG pathway, and the results of the enrichment analysis were reordered according to their weight coefficients. The weight coefficients of each pathway were the sum of the weight coefficients of all targets in a specific pathway.

### Animal Experiments

#### Experimental Animals Mental Animal

Male Kunming mice (*n* = 42) weighing 18-22 g were purchased from Chengdu Dashuo Experimental Animal Co., Ltd. under the experimental animal license SCXK (Sichuan) 2020-030. This study was approved by the Animal Ethics Committee of Shaanxi University of Chinese Medicine.

#### Establishment of the Eczema Model

To establish the eczema model, all groups, except the normal group, were sensitized on the back with 7% DNCB solution. In brief, the back hair in a 2 cm × 2 cm area was removed, and all the groups, except the normal group, were sensitized with 100 µL acetone olive oil solution containing 7% DNCB. After 5 days, all the groups, except the normal group, were challenged with 30 µL acetone olive oil solution containing 1% DNCB on the inside and outside of the right ear. The challenge was repeated three times in two consecutive days. As a control, mice in the normal group were smeared with the same amount of acetone olive oil solution on the left ear.

#### Experimental Grouping and Administration

The mice were adaptively fed for 3 days and randomly divided into six groups (*n* = 7/group): normal group, model group, positive drug group, low-dose chamomile group (concentration 0.15%), medium-dose group (concentration 0.3%), and high-dose group (concentration 0.5%). Each group was reared in separate cages. The aforementioned groups were treated via skin smearing on the eczema site twice a day for 14 consecutive days. The German chamomile volatile oil group was treated with 80 µL volatile oil each time in the positive drug group, and 80 µL olive oil solvent was applied in the normal group and model groups.

#### Mouse Skin Test and Determination of Spleen Index

After the completion of treatment, the skin water content, sebum content, and skin elasticity of the treated area were measured using an intelligent digital display multi-function skin detector (RBX-916). The mice in each group were weighed before sampling, and the spleen was removed after carefully separating the connective tissue around the spleen. After absorbing the residual blood on the organ surface with absorbent paper, the spleen mass was immediately weighed with an analytical balance. The spleen index was calculated as follows: spleen index = (spleen quality (mg))/(experimental mouse quality (g)). Each group was compared with the control group, and the difference was calculated.

#### Hematoxylin–Eosin Staining

Tissue specimens were fixed with 4% paraformaldehyde for 24 h gradient dehydrated with 75, 85, and 95% anhydrous ethanol until transparent and embedded in paraffin, cut into 4-µm-thick tissue sections, and then spread in water at 43°C (Zhang et al., 2020). The tissue slices were baked at a constant temperature and dried in a blast drying box at 60°C for 2-4 h. After dewaxing, the tissue sections were soaked in gradient alcohol and hydrated in distilled water for several minutes. The tissue sections were stained with hematoxylin and eosin. After dehydration, the tissue sections were sealed with xylene, and the changes were observed under a microscope.

#### Toluidine Blue Staining

The paraffin-embedded tissue was sliced (4 µm) (Chen et al., 2020). Each slice was stained with 0.5% toluidine blue dye solution at room temperature for 30 min and rinsed quickly with distilled water. The slices were placed in 0.5% glacial acetic acid, differentiated for several minutes and sealed via alcohol dehydration, rendered transparent with xylene, sealed with neutral gum, and observed under a microscope.

#### Immunohistochemistry

The slices were incubated with 3% hydrogen peroxide for 25 min and incubated with 10% normal goat serum at room temperature for 30 min. Serum was removed, the primary antibody was added, and tissues were incubated overnight at 4°C. The tissues were then washed three times with PBS for 5 min per wash. The secondary antibody was added, and tissues were incubated at room temperature for 50 min. DAB chromogenic solution was used to cover the tissues evenly, and the tissues were sliced into hematoxylin for 2 min and observed under a microscope after dehydration, transparency, and sealing.

#### Enzyme-Linked Immunosorbent Assay

The content of IL-6, TNF-α, and IL-17 in the serum of mice from each group was detected by ELISA, and a standard curve was drawn according to the kit instructions (Jiangsu Meimian industrial Co.,Ltd) (Šutovská et al., 2021). The cytokine content in the serum of each group was detected via the double antibody sandwich method and enzyme labeling instrument.

#### Western Blot Analysis (Gao et al., 2018).

Skin tissues from the aforementioned groups were collected and lysed with RIPA buffer, and the lysed samples were put on ice for 5 min. The supernatant was centrifuged in a cryopreserved centrifuge at 4°C, 12,000 rpm for 10 min. The supernatant was separated as the protein extract. A total of 40 µg protein was separated *via* sodium dodecyl sulfate–polyacrylamide gel electrophoresis (SDS-PAGE) and then transferred to polyvinylidene fluoride (PVDF) membranes. The membranes were sealed with 5% skimmed milk powder and then incubated with antibodies for p38 (Wanleibio, WL00764, China), p-p38 (Wanleibio, WLP1576, China), p65 (Wanleibio, WL01980, China), and p-p65(Wanleibio, WL02169, China). Then, the membranes were washed with Tris-buffered saline + Tween (TBST) and incubated with the secondary antibodies. The membranes were then washed in TBST six times, sprinkled evenly with electrochemiluminescence (ECL) photoluminescence solution, and transferred to a dark box for exposure. The film was scanned, and the optical density was calculated using Gel-Pro Analyzer software. The relative expression value of the target gene (the gray value of the target gene/the internal reference gray value of β-actin) was determined using β-actin as an internal reference.

### Molecular Docking

The active ingredients of German chamomile were selected as ligands, the first five proteins of the prediction pathway were used as targets, and the area where the positive drug ligand was located was the active pocket (Jia et al., 2021). The LibDock module in the discovery studio software was used to molecularly dock the active ingredients with the target protein. After the docking was completed, the established formula was used to calculate the score of each component to screen out the key components of the target.

$$S=Z*\frac{A}{B}.$$

In the formula, S represents the final score of each component, Z represents the score of each active compound, A represents the score of each component obtained by molecular docking, and B represents the score of positive drugs.

### Statistical Analysis

All of the experimental data were statistically analyzed using SPSS 19.0 software. Results are expressed as mean ± standard deviations (SDs). One-way analysis of variance (ANOVA) was applied to determine the statistical significance of the differences between the groups, *p* < 0.05 and *p* < 0.01 were regarded as significantly different.

## Results and Analysis

### Screening of Compounds and Selection of Potential Targets

**FIGURE 1:**
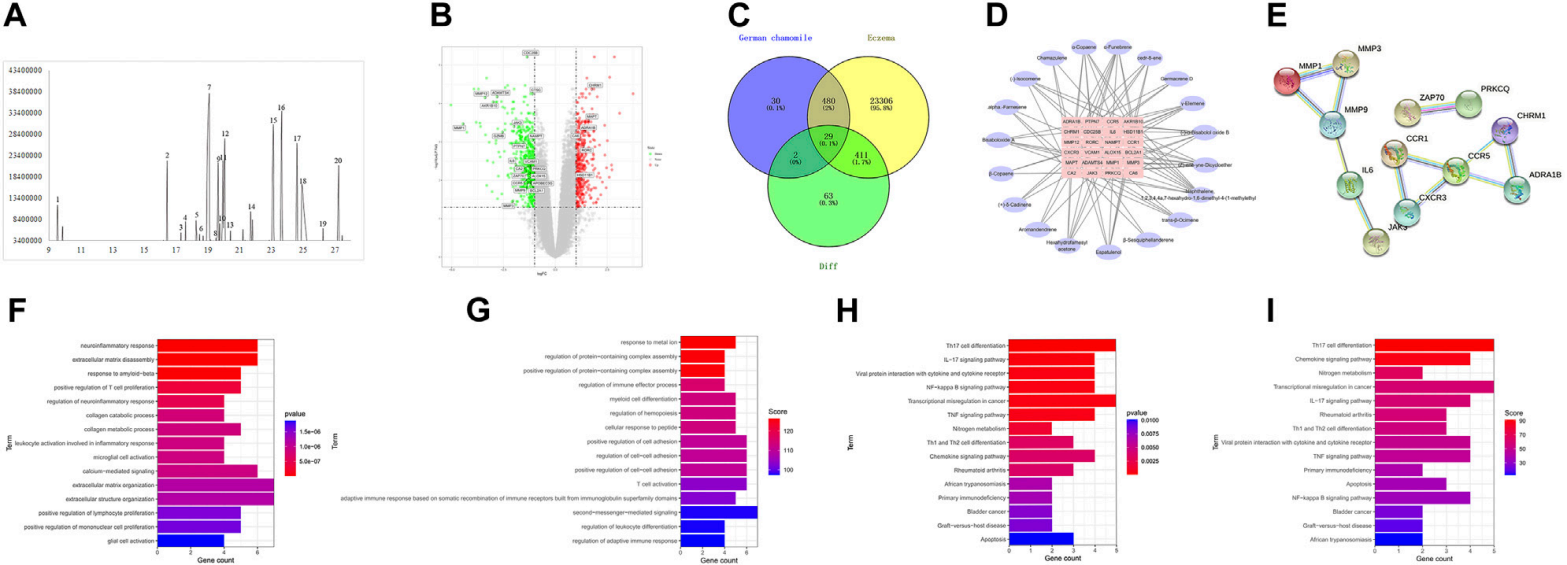
**(A)** Total ion current diagram of volatile oil of German chamomile. **(B)** Eczema differential gene volcano map. **(C)**: Intersection map of German chamomile volatile oil target, eczema target, and differential gene. **(D)** Volatile oil composition of German chamomile target map. **(E)** PPI network diagram of key targets. **(F,H)** Enrichment results of BP and KEGG before sorting, respectively. **(G,I)** Enrichment results after sorting BP and KEGG.

**FIGURE 2:**
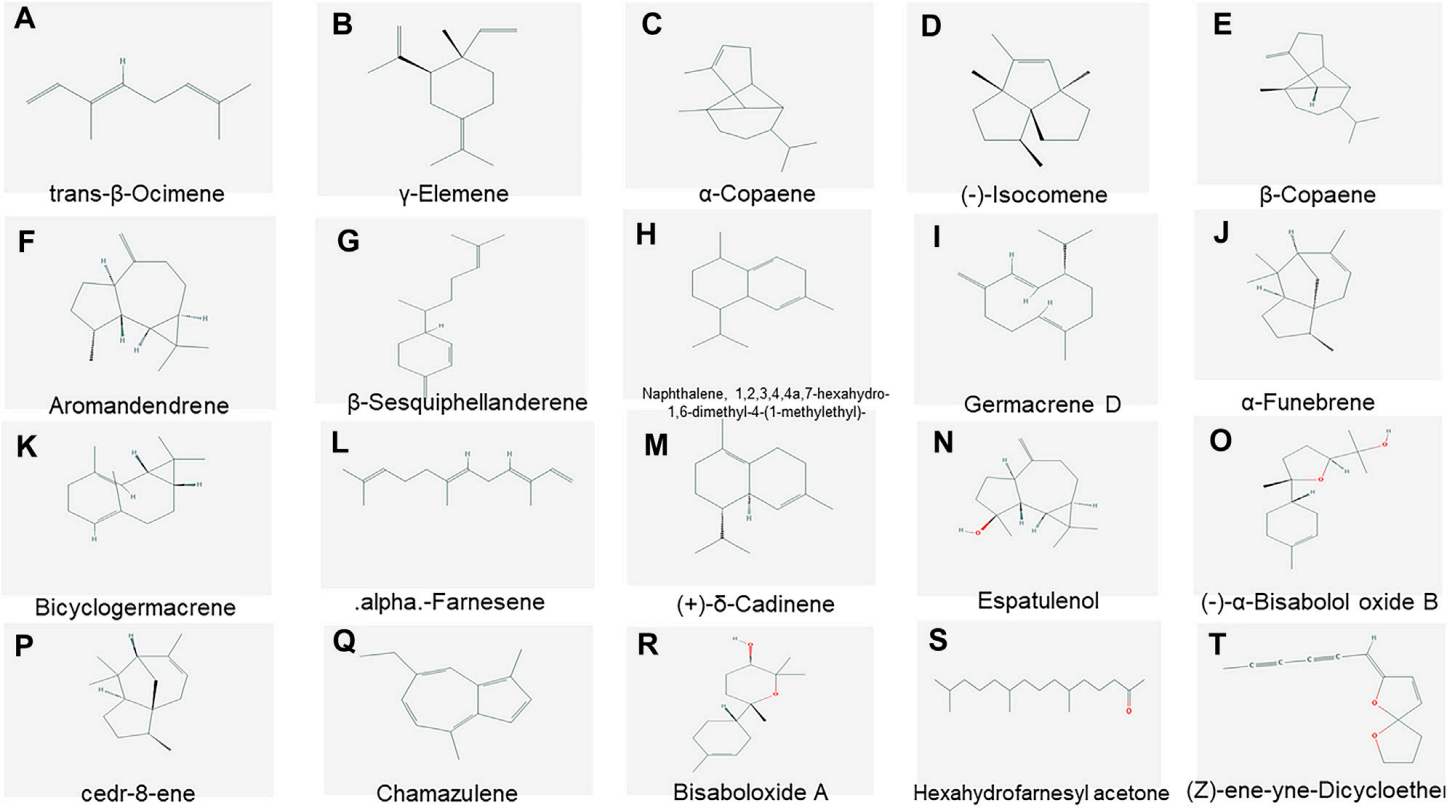
**(A–T)** Chemical structures of 20 active components.

The essential oil of chamomile was analyzed by gas chromatography–mass spectrometry (GC–MS), and the ion mass spectrum of the essential oil was obtained (Figure 1A). By searching the NIST database and combining with the retention index, 23 active components were obtained and 20 qualified components were identified. Specific composition information is shown in Table 1, and specific compound structures are shown in Figure 2. A total of 541 targets of German chamomile volatile oil were obtained using the PubChem database, the Swiss TargetPrediction online target screening platform, and the meta TarFisher database. Additionally, 24,226 eczema targets were obtained from the GeneCards, DisGeNet, OMIM, and CTD databases. Based on the intersection, 509 potential targets of German chamomile volatile oil for the treatment of eczema were obtained.

**TABLE 1:** Qualitative results of volatile oil from German chamomile by GC/MS

No.	Library/ID	CAS	Pct Total	RI
1	Trans-β-ocimene	3779-61-1	2.728	1,049.212
2	γ-Elemene	29873-99-2	3.882	1,343.234
3	α-Copaene	003856-25-5	0.756	1,382.972
4	(-)-Isocomene	065372-78-3	0.943	1,396.072
5	β-Copaene	018252-44-3	2.639	1,427.655
6	Aromandendrene	000489-39-4	0.653	1,448.85
7	β-Sesquiphellandrene	020307-83-9	18.527	1,467.285
8	Naphthalene, 1,2,3,4,4a,7-hexahydro-1,6-dimethyl-4-(1-methylethyl)-	16728-99-7	0.661	1,487.56
9	Germacrene D	023986-74-5	4.835	1,494.01
10	α-Funebrene	50894-66-1	1.452	1,499.08
11	Bicyclogermacrene	100762-46-7	4.822	1,509.228
12	α-Farnesene	000502-61-4	6.573	1,515.053
13	(+)-δ-cadinene	000483-76-1	0.86	1,533.014
14	Espatulenol	006750-60-3	2.127	1,597.092
15	(-)-α-bisabolol oxide B	026184-88-3	9.46	1,672.963
16	Cedr-8-ene	000469-61-4	9.606	1,718.242
17	Chamazulene	000529-05-5	6.398	1,952.009
18	Bisabolol oxide A	022567-36-8	12.071	1,970.864
19	Hexahydrofarnesyl acetone	000502-69-2	0.888	2,043.486
20	(Z)-ene-yne-dicycloether	004575-53-5	6.074	1,906.516

### GEO Chip Differential Gene Verification

The GSE57225 chip and platform data were downloaded from the GEO database. A total of 62 samples were collected, including 17 normal samples and 23 eczema samples. Based on the screening conditions (| logFC | > 1.2 and *p* < 0.05), 560 genes with significant differences were obtained. The upregulated gene expression (red), downregulated gene expression (green), and the volcano plot are shown in Figure 1B. The intersection between differentially expressed genes (DEG) and possible eczema targets of German chamomile volatile oil produced 29 candidate genes, as shown in Figure 1C.

### Network Construction and Analysis

Network pharmacology provides a new perspective on analyzing the effects of drugs. It can analyze network characteristics through the connections and relationships of nodes in biological networks, and further clarify the mechanism of drug action. We analyzed the relationship between the active ingredients and key targets of the volatile oil of German chamomile and then created a “component–target” map with a merge function. We then used Cytoscape 3.7.1 software to visualize the network. The results are shown in Figure 1D. The purple ellipse represents the key components screened out by the volatile oil, the pink rectangle represents the key target points screened out, the node represents the active ingredient, and the edge is used to connect the target to the active ingredient. The greater number of links indicates that the active ingredient or the target is more important in the network.

### German Chamomile–Eczema–Differential Gene PPI Network Construction and Key Target Screening

The 29 DEGs were introduced into STRING to construct a network map, as shown in Figure 1E. Through the screening of the set conditions, the key targets were identified as IL-6, MMP1, MMP3, MMP9, JAK3, CCR1, CCR5, ZAP70, and PRKCQ. The network diagram had 29 nodes with an average degree of freedom of 0.828. The size of the node in the diagram represents the size of the value. The thickness of the edge indicates that the thicker the edge, the greater the combined score value and the greater the interaction between the nodal protein and other proteins.

### GO–BP and KEGG Enrichment Analysis

Through GO–BP enrichment analysis of the selected key targets, 145 biological processes and 16 signaling pathways, were identified (Figures 1F–I). These involved the neuroinflammatory response, extracellular matrix disassembly, response to amyloid-beta, positive regulation of T-cell proliferation chemokine signaling pathway, Th17 cell differentiation, IL-17 signaling pathway, and viral protein interaction with cytokine and cytokine receptor, among other signaling pathways. After recalculating the formula, Th17 cell differentiation was determined to be the most important one.

### Animal Experiment and Efficacy Verification

#### Comparison of the Degree of Inflammation

**FIGURE 3:**
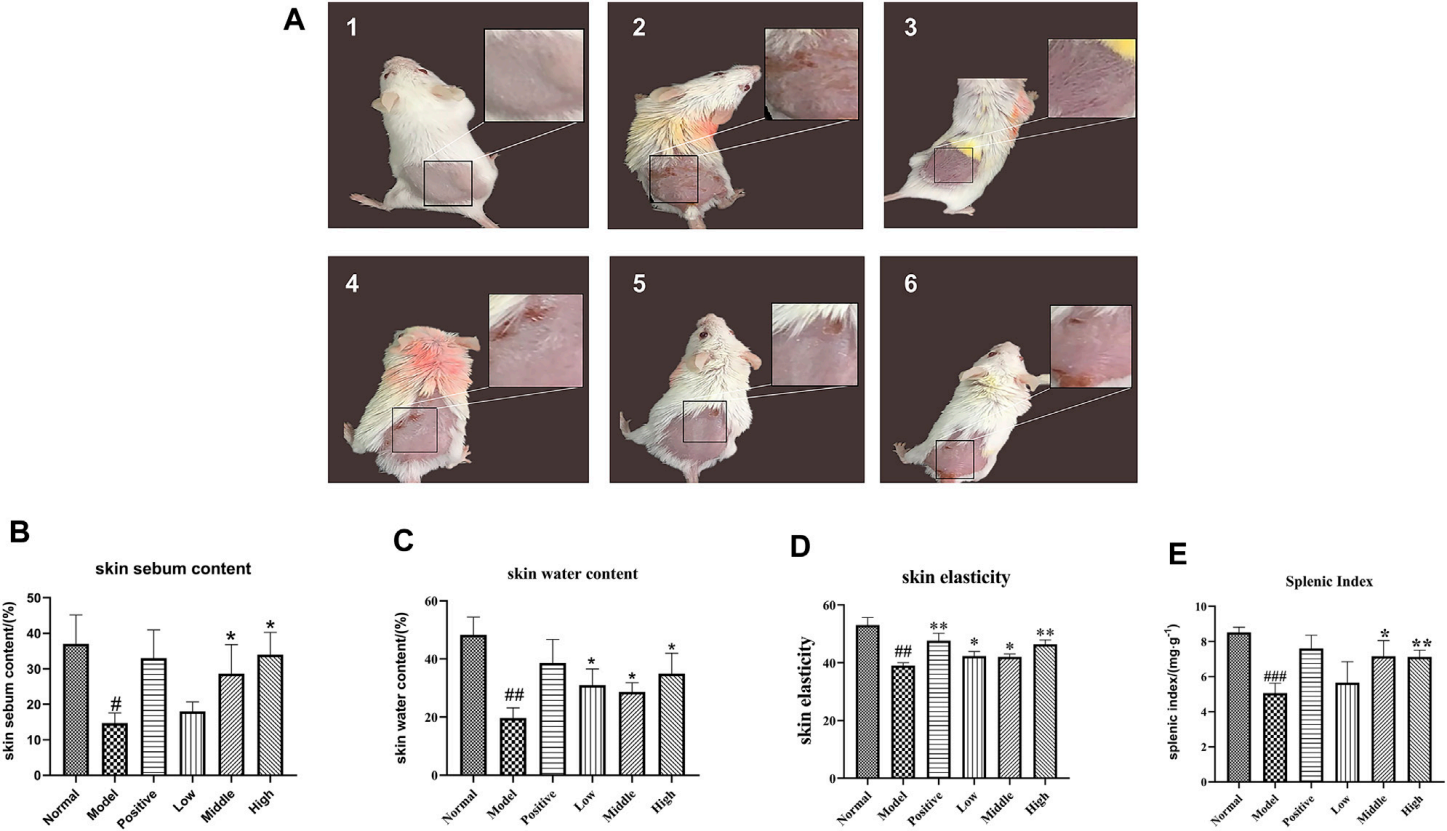
**(A)**: Effects of volatile oil of German chamomile on back inflammation in mice (A1: normal group, A 2: model group, A 3: positive drug group, A 4: low-dose German chamomile group, A 5: middle-dose German chamomile group, and A 6: high-dose German chamomile group). **(B)**: Mice skin sebum content test results. **(C)**: Mice skin water content results. **(D)**: Mice skin elasticity test results. **(E)**: Mice spleen index measurement results (note: data are expressed as mean ± SD (*n* = 6), compared with the normal group #*p* < 0.05, ##*p* < 0.01, compared with the model group *p < 0.05, **p < 0.01).

After DNCB induction, each group exhibited skin erythema, exudation, thickening, a rough surface, and moss-like lesions, suggesting that the model was successful. After treatment with the positive drugs and different doses of German chamomile volatile oil, the exudate, redness, and swelling were reduced. In the positive drug and high-dose German chamomile volatile oil groups, eczema was reduced, as shown in Figure 3A.

#### Skin Test Results

Skin test results showed that when compared with the normal group, the skin sebum content (Figure 3B), the skin water content (Figure 3C), and skin elasticity (Figure 3D) in the model group decreased. Compared with the model group, the skin water content and sebum and skin elasticity of different dose groups increased to different degrees, with significant differences.

#### Comparison of Spleen Index Among Different Groups of Mice

The effect of volatile oil from German chamomile on the immune response was preliminarily evaluated by the spleen index (Figure 3E). The chart shows that the spleen index of the model group was lower than that in the normal group, and the spleen index of different dose groups was higher than that in the model group, with significant differences.

#### H&E Staining Results

**FIGURE 4:**
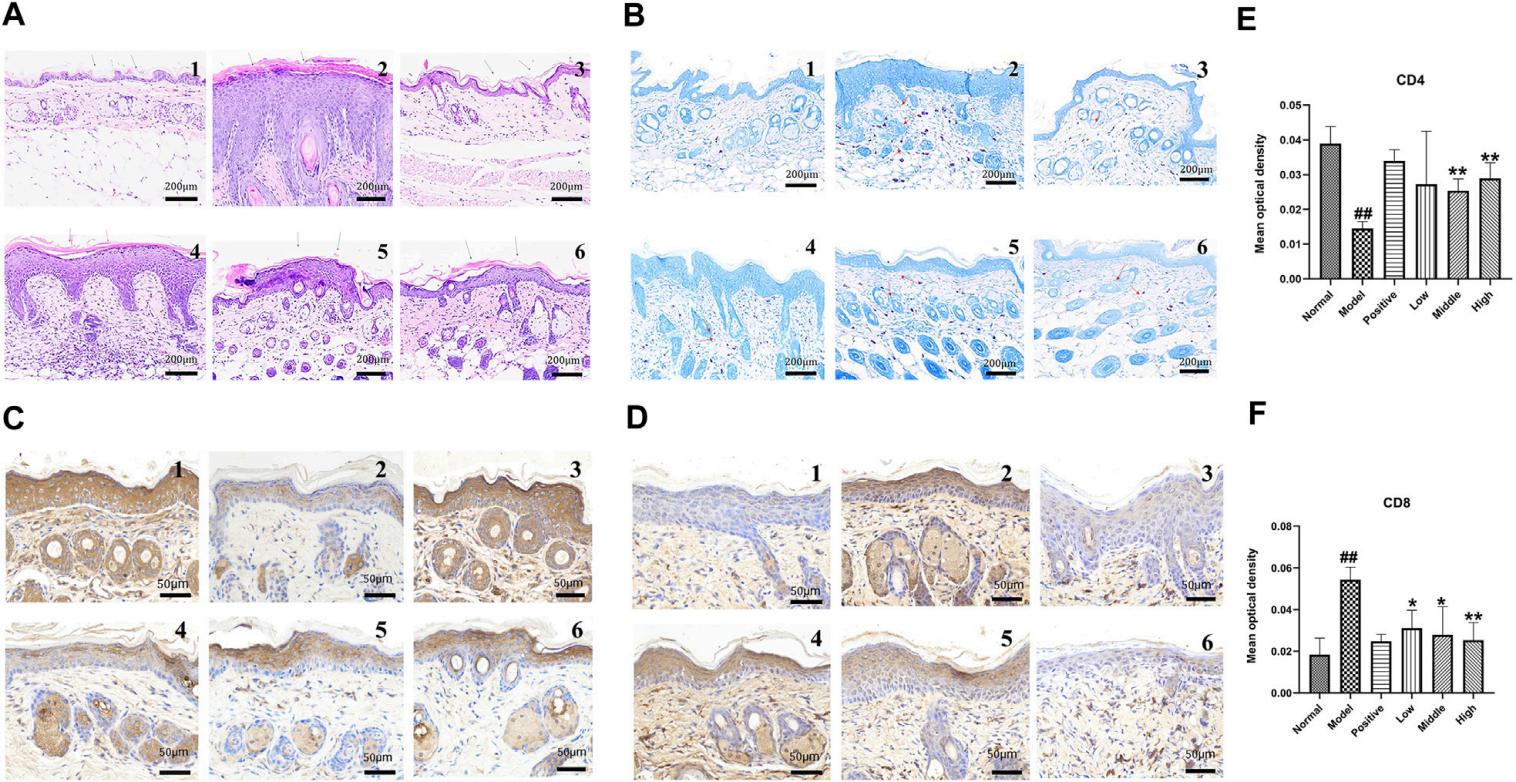
**(A)** H&E staining results of mice skin tissue. **(B)** Mast cell count by toluidine blue staining. **(C)** CD4 cell staining results. **(D)** CD8 cell staining results (note 1: normal group, 2: model group, 3: positive drug group, 4: low-dose group, 5: middle-dose group, and 6: high-dose group). **(E)** Immunohistochemical detection of CD4 cell expression in skin lesions on the back of mice. **(F)** Immunohistochemical detection of CD8 cell expression in skin lesions on the back of mice (note: data are expressed as mean ± SD (*n* = 3) compared with the normal group ##*p* < 0.01, compared with the model group, *p < 0.05, **p < 0.01).

The results of H&E staining (Figure 4A) showed that the structure of the epidermis and dermis in the normal group appeared normal, without edema, congestion, or lymphocyte infiltration. In the model group, the epidermis had proliferated, and the spinous layer was hypertrophic. The dermis showed inflammatory cell infiltration accompanied by vasodilation and hyperemia. In the positive drug group, the epidermis had proliferated slightly, and most of the dermis had recovered. No edema, hyperemia, or lymphocyte infiltration was apparent. The epidermal hyperplasia and cell edema significantly decreased, with the increase in dose in the treatment group.

#### Toluidine Blue Staining Results

Results of toluidine blue staining are shown in Figure 4B. The number of mast cells was low in the normal group and high in the model group. The mast cell number was significantly reduced, in the positive drug group and decreased in a dose-dependent manner in the low, middle, and high German chamomile volatile oil groups.

#### CD4 and CD8 Staining Results

CD4 and CD8 cells are located in the cytoplasm and cell membrane. CD4 and CD8 cells were weakly positive in the skin tissues of the back of mice. Compared with the normal group, the distribution of CD4 cells in the cytoplasm and cell membrane of the skin lesions on the back of the eczema model group was reduced, the staining became lighter, and the positive expression of CD4 cells was reduced (*p* < 0.001, *n* = 3). Compared with the eczema model group, different doses of German chamomile volatile oil increased the expression of CD4 cells to varying degrees, and the medium and high doses were significantly different from the model group (*p* < 0.001, *n* = 3).

Compared with the normal group, CD 8 cells in the skin lesions on the back of mice in the eczema model group showed brownish-yellow particles in the cell membrane and cytoplasm, and the positive expression level of CD8 cells increased (*p* < 0.001, *n* = 3). Compared with the eczema model group, different doses of German chamomile volatile oil caused different degrees of reduction in the number of cells expressing CD8. There were significant differences between the high-dose group and the model group (*p* < 0.001, *n* = 3), as shown in Figures 4C–F.

#### Serum Levels of TNF-α, IL-6, and IL-17

**FIGURE 5:**
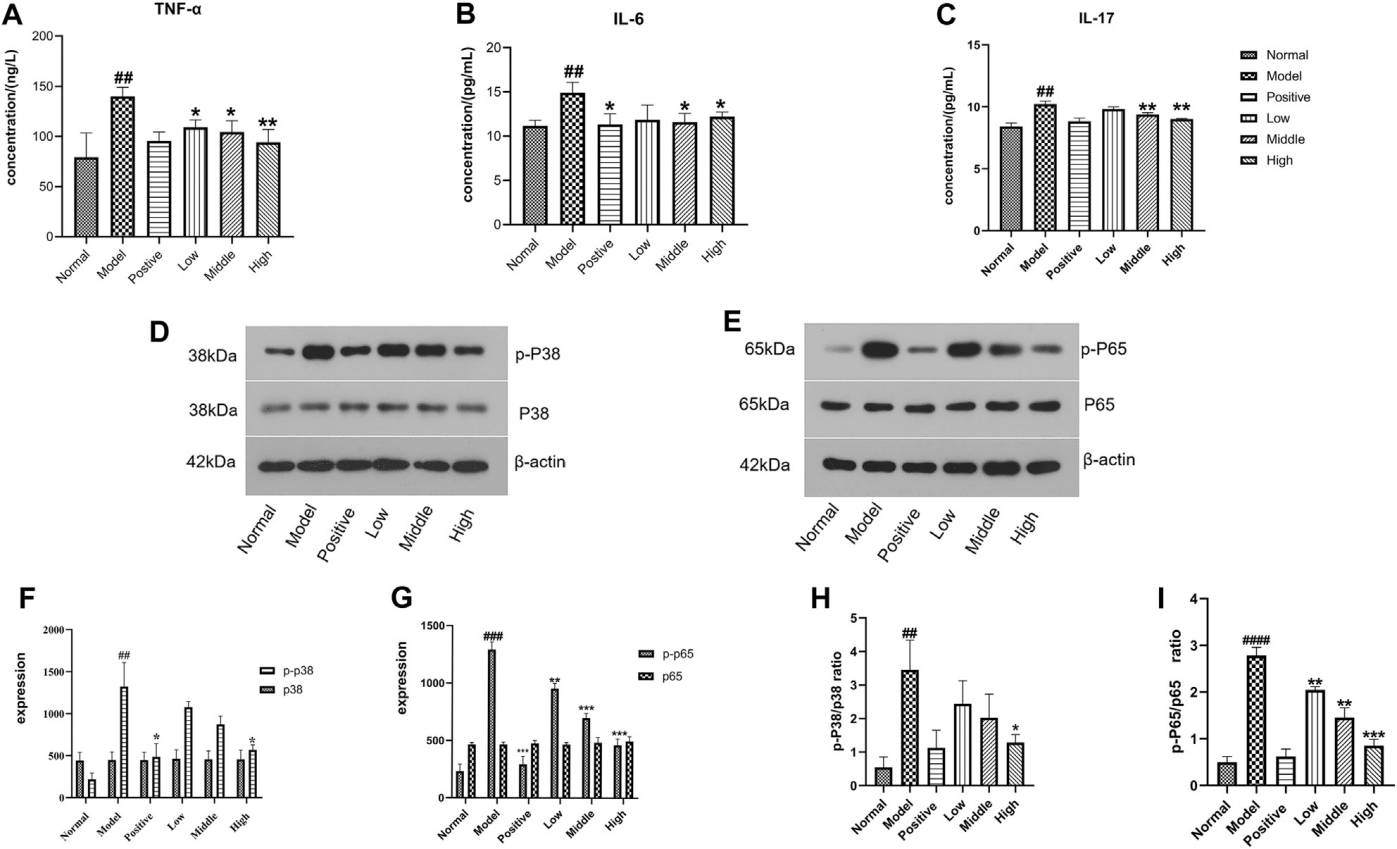
**(A)** ELISA detection of TNF-α. **(B)** the ELISA detection of IL-6. **(C)** ELISA detection of IL-17 (note: the data are expressed as the mean ± SD (*n* = 3), compared with the normal group ##*p* < 0.01, compared with the model group, *p < 0.05, **p < 0.01). **(D–I)** Western blot was used to detect the protein expression levels of p-p38, p38, p-p65, p65,p-p38/p38, and p-p65/p65. **(D,E)** Quantitative protein expression levels of p-p38, p38, p-p65, and p65. **(F,G)** Protein expression level of p38, p-P38, p65, and p-P65. **(H,I)** Ratio of p-P38/p38 and p-P65/p65 in mouse skin lesions. (x¯±s (n = 3), compared with the normal group ###*p* < 0.001, ##*p* < 0.01, compared with the model group ***p < 0.001,*p < 0.05).

Compared with the normal group, serum TNF-α, IL-6, and IL-17 in the eczema model group increased significantly (*p* < 0.001, *n* = 6). Compared with the model group, these factors were reduced in the positive drug group and the German chamomile volatile oil groups. The results are shown in Figures 5A–C.

#### Western Blot Analysis

The expression levels of p-P38 and p-P65 proteins in the positive drug group and the treatment group were lower than those in the model group. Compared with the normal group, the expression of p-P38/p38 and p-P65/p65 protein in the mouse eczema model group was higher than that of the mouse eczema model group (*p* < 0.001, *n* = 3). Compared with the mouse eczema model group, both the treatment group and the positive drug group had reduced protein expression of p-P38/p38 and p-P65/p65 (*p* < 0.001, *n* = 3), which indicates that German chamomile volatile oil can inhibit the MAPK and NF-κB pathways. The results are shown in Figures 5D–I.

### Molecular Docking Verification

**FIGURE 6:**
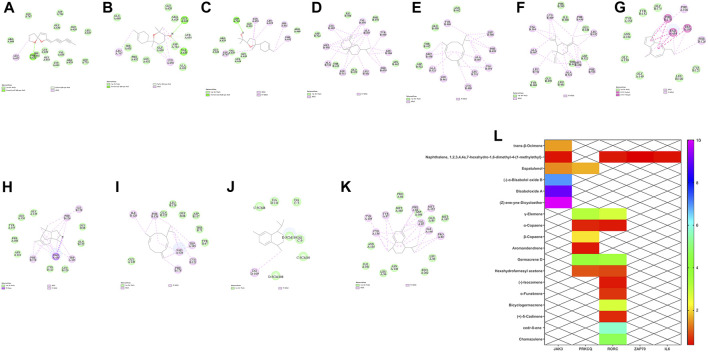
**(A–C)** Results of docking of JAK3 and components (Z)-ene-yne-dicycloether, bisabolol oxide A, (-)-α-bisabolol oxide B. **(D–F)** Results of docking of PKC8 and components germacrene D, γ-elemene, and β-copaene. **(G–I)** Results of docking of RORC target with components germacrene D, chamazulene, and cedr-8-ene. **(J)** IL6 target and component naphthalene, 1,2,3,4,4a, 7-hexahydro-1,6-dimethyl-4-(1-methylethyl)- docking result. **(K)** ZAP70 target and component naphthalene, 1,2,3,4,4a, 7-hexahydro-1,6-dimethyl-4-(1-methylethyl)- docking result. **(L)** Comprehensive heat map display.

The molecular docking results are shown in Figure 6A–K. The molecular docking of the core active component and the target was conducted using Discovery software, and the molecular docking score was calculated using the new weight formula. The higher the score of the component, the higher the importance of the component to the target and the greater the role of the component in the Th17 cell differentiation pathway. The results are shown in the heat map in Figure 6L. These results suggest that chamazulene, bisabolol oxide A, (-)-α-bisabolol oxide B, and γ-elemene may be key active components of German chamomile volatile oil in the treatment of eczema.

### Mechanism Description

**FIGURE 7:**
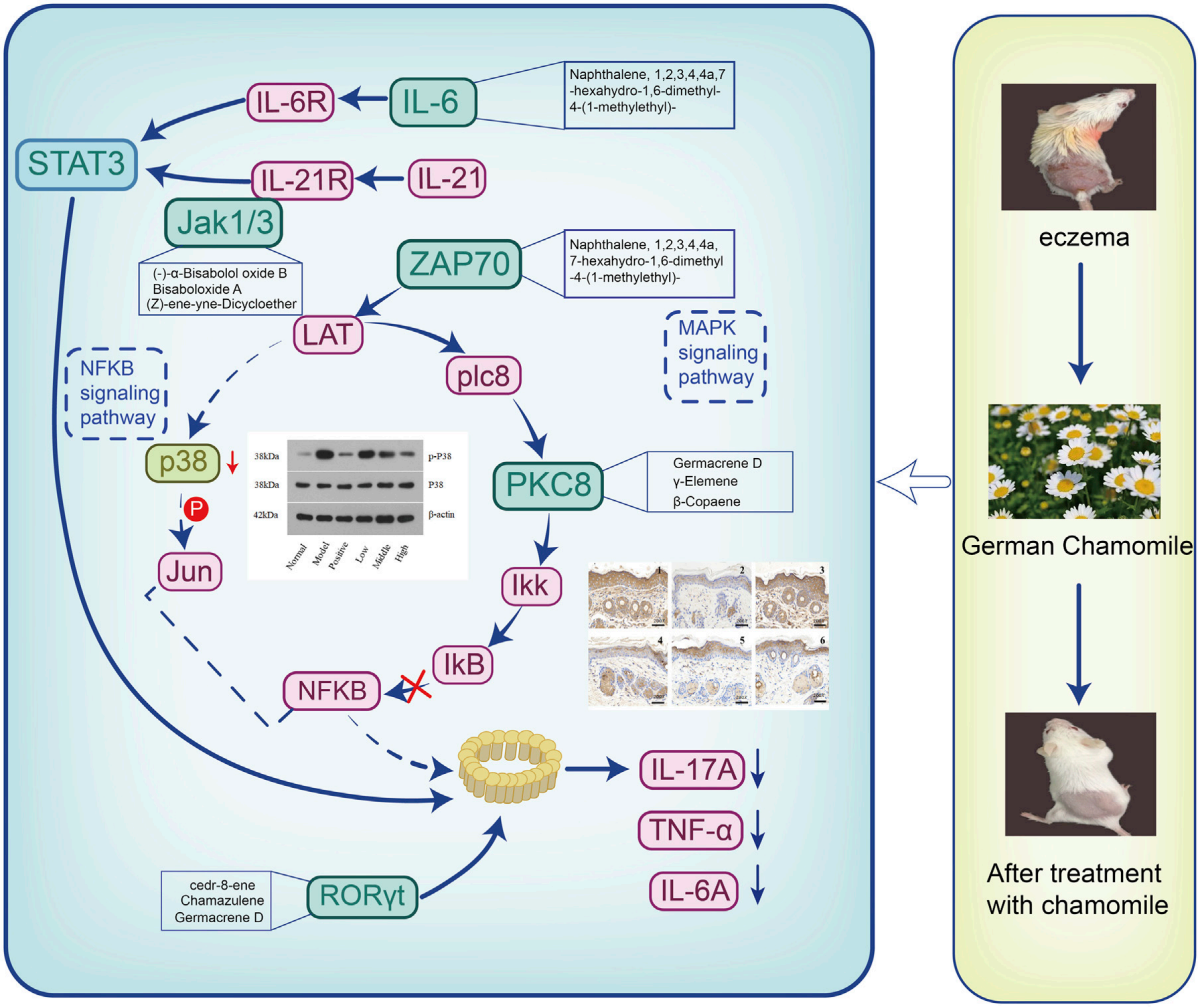
Mechanism display.

We used the dinitrochlorobenzene (DNCB) method to establish a mouse eczema model. The models were treated with low, medium, and high doses of German chamomile volatile oil. During the experiment, the mice were observed. Skin conditions include sebum content, water content, and elasticity index. The serum levels of inflammatory factors IL-6, IL-17, and TNF-α in different groups of mice were detected by ELISA, and the inflammatory cells, lymphocytes, and mast cells in the skin lesions of the mice were observed by H&E staining and toluidine blue staining. To detect the level of CD4 and CD8 cells of lymphocytes immunohistochemistry was used, and finally, to show the protein expression levels of p38, p65, p-P38, and p-P65 in the skin lesions Western blot was used. Combined with the results of each experiment, we believe that the mechanism of action of German chamomile volatile oil in the treatment of eczema may be German chamomile volatile oil can prevent the differentiation of CD4^+^ cells into Th17 cells by regulating the lymphatic subsets of T cells; inhibiting the activation of MAPK and NF-κB pathways; reducing the secretion of inflammatory factors IL-6,IL-17, and TNF-α; and thereby reducing the inflammatory response (Figure 7).

## Discussion

In the treatment of eczema, German chamomile volatile oil is administered transdermally. Therefore, our research included the water–oil distribution coefficient (log P) and composition content as key parameters in predicting absorption into blood. We have established “weight coefficients” that can be compared with the signaling pathways enriched by traditional network pharmacology. This can be used to reassess the results of biological enrichment analysis to determine key pathways. Before and after the ranking the contribution scores of the “weight coefficient,” we found that the top five pathways enriched by traditional network pharmacology were Th17 cell differentiation, the IL-17 signaling pathway, viral protein interaction with cytokine and cytokine receptor, the NF-κB signaling pathway, and transcriptional dysregulation in cancer. Following the calculation using the new weighting coefficient, the top five enriched pathways were the Th17 cell differentiation, chemokine signaling pathway, nitrogen metabolism, transcriptional misregulation in cancer, and IL-17 signaling pathway. Through literature review in combination with sequencing the pathways, we determined that Th17 cell differentiation is a key pathway for German chamomile volatile oil in the treatment of eczema.

Studies (Liu et al., 2019) have shown that the occurrence of dermatitis and eczema is related to an imbalance in T-cell lymphatic subsets (Th1/Th2). TNF-α secreted by Th1 cells induces the expression of ICAM-1 and L-selectin, causing T cells and macrophages to infiltrate a large number of T cells and macrophages to the inflammatory site. With inflammatory stimulation, monocytes, macrophages, and endothelial cells will release Th2 cell IL-6. The excessive secretion of Th2 type cytokines is an important factor leading to eczema, and the excessive secretion of Th1-type cytokines aggravates the disease (Zhou et al., 2019). The level of Th17 in the peripheral blood of patients with eczema is significantly higher than that in normal people (Fan et al., 2019), and these cells secrete a large number of inflammatory cytokines that induce tissue inflammation. IL-17 secreted by Th17 cells can stimulate skin keratinocytes, increase the secretion of pre-inflammatory cytokines, and expand skin inflammation and tissue destruction (Xu and Lv, 2020). Research studies had shown that the levels of TNF-α, IL-6, and IL-17 cytokines in the serum of mice treated with German chamomile volatile oil were lower than those in the model group, indicating that the decrease in TNF-α, IL-6, and IL-17 may be one anti-inflammatory mechanism. After recognition by cell surface IL-17 receptors, IL-17 can activate downstream signaling pathways such as NF-κB and MAPK, leading to the expression of pro-inflammatory chemokines and cytokines.

In multicellular organisms, especially mammalian cells, multiple signaling pathways often must work together to accurately complete cell functions (Yong and Xiao-Wei, 2000). The MAPK pathway is a common intersection pathway for signal transduction pathways affecting cell proliferation, differentiation, transformation, and apoptosis (Lei et al., 2020). Some drugs participate in the activation, proliferation, and migration of immune cells via the MAPK signaling pathway to alleviate skin lesions due to AD allergic disease (Pfalzgraff et al., 2016). Oxidative damage in skin cells may be reduced via the use of a p38 inhibitor, which inhibits the downregulation of IL-6. Effective substances may attenuate the expression of NF-κB in keratinocytes and induce thromboxane regulation, which is related to the downregulation of inflammatory cells (Chang et al., 2017; Sur et al., 2019). NF-κB plays an important role in regulating T-cell autoimmunity and inflammation. The transcription factor p65 is a member of the NF-κB family, which can induce the production of pro-inflammatory chemokines induced by the TNF-α/IFN-γ *in vitro* and can also induce an allergic inflammatory response. p38MAPK is the upstream kinase of NF-κB. It is a stress-induced protein kinase. It is activated under the action of inflammatory factors and oxidative stress and enters the nucleus from the cytoplasm to further induce NF-κB activation, which eventually leads to the production of a large number of inflammatory factors.

In this study, the model group showed hyperplasia of the damaged epidermis, hypertrophy of the spinous layer, infiltration of a large number of mast cells, infiltration of inflammatory cells in the dermis, and obvious eczema-like manifestations such as vasodilation. In the positive drug group and groups treated with different doses of German chamomile volatile oil, hyperkeratosis was inhibited and inflammatory cell infiltration in the eczema model epidermis was reduced. Also, the water content and lipid content and elasticity of the skin increased, suggesting a similar effect of volatile oil of German chamomile and positive drugs. In T lymphocytes, CD4^+^ cells assist macrophages, B lymphocytes, and killer T cells, whereas CD8^+^ cells have a direct killing effect on antigen-carrying target cells. CD4^+^ and CD8^+^ regulate each other to maintain immune balance. The immunohistochemistry results showed that after treatment with volatile oil, the positive expression of CD4^+^ in the skin lesions on the backs of mice increased, and the positive expression of CD8^+^ decreased, confirming that it can reduce inflammation and infection by improving the body’s immune level. Serum levels of IL-6, TNF-α, and IL-17 in the model group were higher than in the normal group. Compared with the model group, positive drugs and German chamomile volatile oil reduced the content of inflammatory factors in the serum. Additionally, this study showed that the protein expression of P-p38/p38 p-p65/p65 increased after modeling, indicating that by activating the MAPK and NF-κB pathways, it promotes the differentiation of CD4^+^ cells into Th17 cells to produce an inflammatory response, where treatment with the German chamomile volatile oil decreased their expression, suggesting the inhibition of the MAPK signaling pathway via the prevention of p38 phosphorylation (Shen et al., 2020). We conclude that German chamomile may reduce inflammation by regulating Th17 cell differentiation, increasing IL-17 and further affecting the MAPK and NF-κB pathways.

To furthe determine the key active components in German chamomile volatile oil affecting the Th17 cell differentiation pathway, we selected the five key targets, namely, JAK3, RORC, PRKCQ, ZAP70, and IL6, and their corresponding active components and positive drugs as ligands. Molecular docking was used to verify the interaction between the main active components of German chamomile volatile oil and potential targets. Using the combination of ligand fraction, positive drug fraction, and component weight, a new formula for judging components was obtained. The results showed that chrysanthemum, red myrrh alcohol A, red myrrh alcohol B, and γ-elemene may be the key components of German chamomile volatile oil in the treatment of eczema.

## Data Availability

Publicly available datasets were analyzed in this study. These data can be found here: Gene Expression Omnibus database GSE57225 chip.

## Abbreviations

BP, biological process; DEG, differentially expressed genes; DL, drug-like; ECL, electrochemiluminescence; GC-MS, gas chromatography–mass spectrometer; GEO, gene expression omnibus database; GO, gene ontology; HE, hematoxylin–eosin staining; IL-6, interleukin 6; IL-17, interleukin 17; JAK3, Janus kinase 3; KEGG, Kyoto Encyclopedia of Genes and Genomes; MMP1, matrix metalloproteinases 1; NF-κB, nuclear factor kappa-beta; OB, oral bioavailability; PPI, protein–protein interaction networks; PRKCQ, protein kinase C; PVDF, polyvinylidene fluoride; RORC, (human) recombinant protein C; SDS-PAGE, sodium dodecyl sulfate–polyacrylamide gel electrophoresis; SMILES, simplified molecular input line entry specification; TBST, Tris-buffered saline + Tween; TNF-α, tumor necrosis factor alpha; ZAP70, zeta-chain 70.
